# The PEX7-Mediated Peroxisomal Import System Is Required for Fungal Development and Pathogenicity in *Magnaporthe oryzae*

**DOI:** 10.1371/journal.pone.0028220

**Published:** 2011-12-14

**Authors:** Jaeduk Goh, Junhyun Jeon, Kyoung Su Kim, Jongsun Park, Sook-Young Park, Yong-Hwan Lee

**Affiliations:** Department of Agricultural Biotechnology, Center for Fungal Genetic Resources, Center for Fungal Pathogenesis, Center for Agricultural Biomaterials, Plant Genomics and Breeding Institute, Research Institute for Agriculture and Life Sciences, Seoul National University, Seoul, Korea; University of Wisconsin – Madison, United States of America

## Abstract

In eukaryotes, microbodies called peroxisomes play important roles in cellular activities during the life cycle. Previous studies indicate that peroxisomal functions are important for plant infection in many phytopathogenic fungi, but detailed relationships between fungal pathogenicity and peroxisomal function still remain unclear. Here we report the importance of peroxisomal protein import through PTS2 (Peroxisomal Targeting Signal 2) in fungal development and pathogenicity of *Magnaporthe oryzae*. Using an *Agrobacterium tumefaciens*-mediated transformation library, a pathogenicity-defective mutant was isolated from *M. oryzae* and identified as a T-DNA insert in the PTS2 receptor gene, *MoPEX7*. Gene disruption of *MoPEX7* abolished peroxisomal localization of a thiolase (MoTHL1) containing PTS2, supporting its role in the peroxisomal protein import machinery. *ΔMopex7* showed significantly reduced mycelial growth on media containing short-chain fatty acids as a sole carbon source. *ΔMopex7* produced fewer conidiophores and conidia, but conidial germination was normal. Conidia of *ΔMopex7* were able to develop appressoria, but failed to cause disease in plant cells, except after wound inoculation. Appressoria formed by *ΔMopex7* showed a defect in turgor generation due to a delay in lipid degradation and increased cell wall porosity during maturation. Taken together, our results suggest that the MoPEX7-mediated peroxisomal matrix protein import system is required for fungal development and pathogenicity *M. oryzae*.

## Introduction

Peroxisomes are single-membrane organelles present in most eukaryotes, which compartmentalize a variety of enzymes involved in diverse biochemical processes, necessary for life [1]. Important functions of peroxisomes include fatty acid beta-oxidation, glyoxylate cycle, and metabolisms of cholesterol, amino acids antibiotics, and reactive oxygen species, as well as methanol oxidation, in different types of cells and environmental conditions [2]. The improper functioning or regulation of peroxisomes are known to cause detrimental effects leading to human disorders such as Zellweger syndrome and adrenoleukodystrophy, a severe neurodegenerative disease [3], [4].

Peroxisome proteins can be classified into three groups, biogenesis proteins called peroxins (PEXs), matrix proteins and membrane proteins, depending on their role or localization [5]. Matrix proteins are translocated to peroxisomes by a complex matrix protein import system consisting of several PEXs, while peroxisome membrane proteins are inserted into membranes through PEX3, PEX19 and PEX16 [6]. The process of peroxisomal matrix protein import may be depicted into four steps: (1) binding of receptors to matrix proteins; (2) docking to the peroxisome membrane; (3) translocation into the peroxisome matrix; (4) recycling of the receptors [2]. Most peroxisomal matrix proteins have either a peroxisomal targeting signal (PTS) 1 tripeptide sequence (S/A/C)(H/R/K)(I/L/M) at the C-terminus, or a PTS2, nonapeptide sequence (R/K)(L/V/I)X5(H/Q)(L/A) at the N-terminus, which are recognized by the PTS1 receptor PEX5, and the PTS2 receptor PEX7, respectively [1]. In some cases, PTS receptors require other co-receptors. The PTS cargo–receptor complex then enters the peroxisome matrix using the same docking system, but with separate matrix proteins, and is released to the cytosol for recycling of receptors through ubiquitination [2].

Fatty acid degradation, a well-known form of peroxisomal metabolism, was elucidated in the fungus *Aspergillus nidulans*, by Hynes *et al.* [7], [8] while Shen and Berger [9] described differences in fungal fatty acid metabolism, depending on the types of fatty acid and fungal species. In addition, peroxisomal functions are revealed to play diverse roles in sexual reproduction in *Podospora anserina* [10], secondary metabolism of penicillin in *Penicillium chrysogenum* [11], asexual reproduction in *P. chrysogenum* [12], formation of woronin bodies in *Neurospora crassa* [13] and glucose metabolisms in *Cryptococcus neoformans* [14]. In phytopathogenic fungi *Magnaporthe oryzae* and *Colletotrichum lagenarium*, peroxisome functions are important factors in fungal pathogenicity on plant leaves [15], [16].

*Magnaporthe oryzae*, a heterothallic ascomycete fungus, is a causal pathogen of the rice blast, which is one of the most destructive diseases in cultivated rice [17]. Infection by this fungus begins with spore dissemination. Once spores adhere to the plant surface, they initiate germination. Upon recognition of environmental cues such as surface hydrophobicity, the tip of a germ tube develops into a specialized infection structure called an appressorium [18]. During appressorium maturation, a turgor pressure of up to 8 MPa is generated within the appressorium through accumulation of high concentrations of glycerol. The fungus then converts the turgor pressure into mechanical force that can breach the plant cuticule by elaborating a penetration peg. Once the fungus accesses plant tissues, it quickly colonises host cells, leading to development of visible symptoms. In the infection process, several cellular events occurring in or on appressoria, including melanin deposition, glycerol accumulation, and cell wall integrity are known to be prerequisites for turgor pressure-mediated pathogenic development [19], [20]. Glycerol is a major osmolite used to generate turgor pressure, which is generated from glycogen, lipids and carbohydrates such as trehalose [21]. During pathogenesis, peroxisomal functions are known to play a key role in lipolysis, which is essential for turgor generation and cell wall biosynthesis, using the peroxisomal end product acetyl-CoA for host invasive growth of *M. oryzae* [22].

Until now, studies on peroxisomal functions of phytopathogenic fungi have focused on abolishing import of matrix proteins carrying both PTS1 and PTS2, so that only the whole peroxisome functions in pathogenicity were reported. However, function of each peroxisome import system during pathogenesis is unclear in *M. oryzae*. For a more complete picture of the contribution of peroxisomes to fungal pathogenicity, we undertook a functional characterisation of *PEX7* and its cargo proteins in *M. oryzae*. Study of the PTS2-mediated import system by PEX7 in this paper is important for both characterization of the roles of PEX7 in pathogenesis and for inferring functions of other PTS import systems.

In previous research, we identified a *M. oryzae* insertional mutant, ATMT0060C3, in which a T-DNA is inserted into a gene encoding a putative PTS2 receptor (*MoPEX7*) [23]. *MoPEX7* is an orthologue of *PEX7*, which was first identified by one of oleic acid non-utilization mutants in *Saccharomyces cerevisiae* [24]. To comparatively characterize functional roles of MoPEX7, we also generated *MoPEX7* gene deletion mutants in this study. Our data revealed that *MoPEX7* is required for short-chain fatty acid metabolism and pathogenicity, independently of PTS1in *M. oryzae*. In particular, MoPEX7 plays an important role in turgor generation during appressorium maturation. To further understand cellular roles of PEX7, we identified putative PTS2 proteins that might be translocated into peroxisomes by PEX7-mediated import system, among which the translocation of MoTHL1 (a 3-ketoacyl CoA thiolase) to peroxisomes was exhibited to be mediated by MoPEX7. Taken together, our results suggest that the MoPEX7-mediated peroxisomal matrix protein import system is required for fungal development and pathogenicity of *M. oryzae*.

## Results

### Identification of a *MoPEX7* gene encoding a PTS2 receptor

Previously, we performed a random insertional mutagenesis using *Agrobacterium tumefaciens*-mediated transformation in *M. oryzae* KJ201 strain to identify novel genes involved in fungal pathogenicity [23]. A transformant, ATMT0060C3, was identified as a mutant lacking pathogenicity to rice. Southern DNA hybridization analysis suggested that ATMT0060C3 has multiple T-DNAs in a single locus (Figure S1B). TAIL-PCR and inverse PCR showed that ATMT0060C3 contained T-DNA insertion into the second exon region of MGG_01481.6, designated *MoPEX7* (Figure S1A). *MoPEX7* encodes a protein with high similarity to Peroxin7 (PEX7) (Figure S2).

A BLAST search against the NCBI database suggests that MoPEX7 is an ortholog to the PTS2 receptor NcPEX7 of *N. crassa* [25]. Sequencing of MoPEX7 cDNA verified that the *MoPEX7* encoding 345 amino acids has identical sequence and gene structure as annotated in *Magnaporthe* database (http://www.broad.mit.edu/annotation/fungi/magnaporthe/). No other predicted open reading frames (ORFs) similar to MGG_01481.6 were present in the *M. oryzae* genome, as confirmed by Southern hybridization analysis. Phylogenetic analysis showed that MoPEX7 is more closely related to orthologues from pezizomycotina than those from yeast species and basidiomycota (Figure S2). Most PEX7 proteins including MoPEX7 have six WD-40 repeats (IPR001680), while a PEX7 homologue in *Fusarium graminearum* has five WD-40 repeats. Despite the different WD-40 repeat number, MoPEX7 was most closely related to PEX7 in *F. graminearum*. Phylogenetic analysis indicated that PEX7 orthologues are well conserved in fungi and show evolutionary changes at the sequence level but not structural level.

### Genome-wide *in silico* identification of PEXs and PTS2-containing proteins in *M. oryzae*

For better understanding of peroxisomal biogenesis, we searched for peroxins in *M. oryzae* and compared them with those in *A. nidulans, S. cerevisiae, Magnaporthe poae and Gaeumannomyces graminis* [26], [27]. In total, 24 peroxins, including PTS1 and PTS2 receptors Pex5 and Pex7, respectively, were identified in *M. oryzae*. Among them, 15 peroxins are related to matrix protein import, three are related to peroxisomal membrane biogenesis and the other seven are related to peroxisome proliferation in *M. oryzae* (Table S1). Distribution of peroxins in *M. oryzae* is more similar to those of fungi belonged to pezizomycotina than *S. cerevisiae*. In the case of the co-receptor of PEX7, PEX20 exists in pezizomycotina, while PEX18 and PEX21 do that function in yeast species. Despite differences in the peroxin system in fungi, PTS receptors are highly conserved compared to other peroxins, which indicates that PTS receptors have a central role in the matrix protein import system. Interestingly, *M. poae* has no orthologs of *PEX2* and *PEX12* in genome unlike other pezizomycotina although previous studies showed that these proteins are conserved in fungi and humans [26]. It is not clear that PEX10 does not form a complex with PEX2 and PEX12 in *M. poae*, or better coverage and annotations are needed for *M. poae* genome.

In an attempt to understand the physiological functions of MoPEX7, we identified PTS2-containing 65 peroxisomal matrix proteins from the *M. oryzae* genome (Table S2). The GO term analysis of the proteins showed that they could be involved in metabolism (35.38%) and cellular processes (23.08%). More than half of the peroxisomal matrix proteins are homologous to characterized genes in other fungi. For example, MGG_10700.6 and MGG_09512.6 show high homology with POT1 in *Yarrowia lipolytica* and FOX3 in *S. cerevisiae*, which are 3-ketoacyl CoA thiolases containing PTS2. These facts suggest that functional roles of PEX7 can be inferred from PTS2-containing peroxisomal matrix proteins.

### Targeted gene deletion of *MoPEX7*

**Figure 1:**
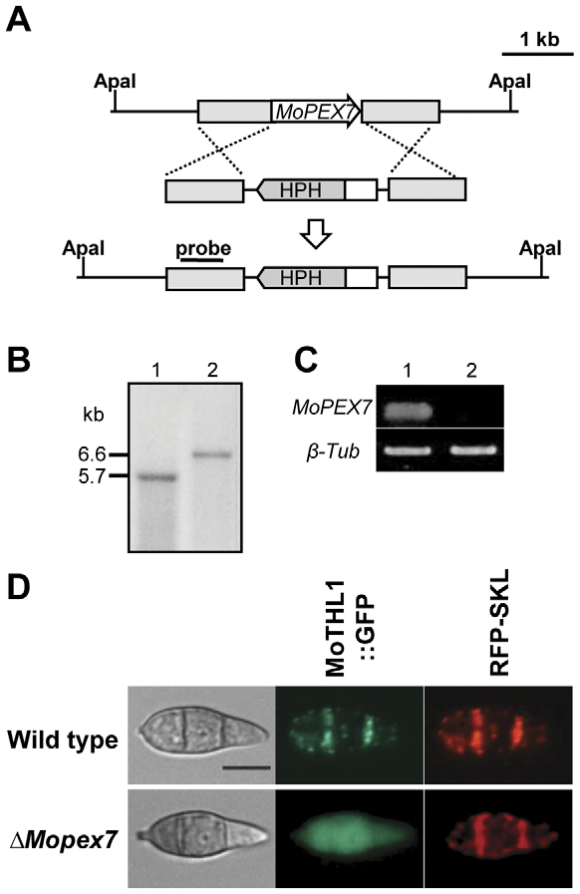
Identification of MoPEX7^T-DNA^ and deletion of *MoPEX7*. (A) Knockout strategy using double-joint PCR. (B) Southern hybridization of *ΔMopex7*. Genomic DNA was digested with *Apa*I. About 1 kb upstream flanking region of *MoPEX7* was used as probe. Lane 1, wild-type strain KJ201; lane 2, *ΔMopex7*. (C) *MoPEX7* gene expression in the wild type and *ΔMopex7* with RT-PCR. Lane 1, wild type; lane 2, *ΔMopex7*. (D) Subcellular localization of the MoTHL1::GFP fusion protein. MoTHL1::GFP containing peroxisome targeting signal 2 was localized in the peroxisome in the wild-type strain KJ201. RFP-SKL (PTS1) was used as positive control. Bar = 10 µm.

ATMT0060C3 (MoPEX7^T-DNA^) was not pathogenic in rice, suggesting that the *MoPEX7* gene is essential for pathogenicity. To ensure that loss of pathogenicity is linked to observed mutation in the transformant, we conducted targeted gene deletion for the *MoPEX7* gene. Using a double-joint PCR strategy, a linear knockout construct with a hygromycin cassette as selectable marker was generated and transformed into the fungal protoplasts of the wild type strain KJ201 (Figure 1A). The resulting hygromycin-resistant transformants were selected using PCR-based screening. Southern hybridization showed that wild type strain KJ201 had a 5.8-kb *Apa*I fragment, while the deletion mutant had a 6.7-kb *Apa*I fragment when probed with the 5′ flanking region of *MoPEX7* (Figure 1B). The transcript of *MoPEX7* was not detectable in reverse transcription (RT)-PCR analysis, supporting that this is the correct gene deletion event in the mutants (Figure 1C).

### MoPEX7 functions as a PTS2 receptor

During peroxisome biogenesis, peroxisomal matrix proteins are imported through two independent pathways. Most peroxisomal matrix proteins have either a PTS1 at the C-terminus, or a PTS2 at the N-terminus. The PTS1 proteins are translocated into the peroxisome by PEX5, and the PTS2 proteins by PEX7 and their co-receptors [1]. To find whether MoPEX7 mediates import of matrix proteins into peroxisomes, we tagged one candidate PTS2 protein, MoTHL1 (MGG_10700.6), with green fluorescent protein (GFP) at the C-terminus and introduced it into both the wild type strain KJ201 and *ΔMopex7*. As a control, we also tested the localization of red fluorescence protein with the PTS1 tripeptide SKL at the C-terminus, which was designated as RFP-SKL. In the wild type KJ201, just like localization of RFP-SKL, many punctate fluorescent spots were observed in spores, indicating localization of MoTHL1::GFP to the peroxisome (Figure 1D). In contrast, in *ΔMopex7*, MoTHL1::GFP was not localized to the peroxisome, resulting in distribution of fluorescence in the cytoplasm, while localization of RFP-SKL was indistinguishable between *ΔMopex7* and KJ201. These results indicate that MoPEX7 functions as a PTS2 receptor, independently of the PTS1 import system, to translocate PTS2 proteins from the cytoplasm into the peroxisome in *M. oryzae*.

### MoPEX7 is required for conidiation

**Figure 2:**
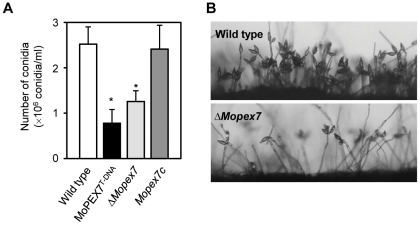
Conidiation and conidiophore differentiation. (A) Conidiation after 10 days post inoculation on oatmeal agar. (B) Conidiophore differentiation.

Conidiation in *ΔMopex7* was dramatically reduced. To further analyze the role of MoPEX7 in conidiation of *M. oryzae*, the wild type and the *ΔMopex7* mutant were grown on oatmeal agar media, and their conidia qualitatively and quantitatively determined. The morphology of conidia produced by *ΔMopex7* was indistinguishable from those of the wild type, but the number of conidia produced by *ΔMopex7* was significantly reduced compared to the wild type. MoPEX7^T-DNA^ produced 30% and *ΔMopex7* produced 50% conidia compared with the wild type (Figure 2A). To clarify the reason for the reduction in conidiation, conidiophore differentiation was observed (Figure 2B), which showed that the mutant develops fewer conidiophores than the wild type. In addition, *ΔMopex7* appears to produce more aerial mycelium than the wild type. But, the mutant conidia retain the ability to germinate and develop appressoria (data not shown). These results suggest that *MoPEX7* is required for conidiophore differentiation and, subsequently, conidiation.

### MoPEX7 is essential for the pathogenicity on rice

**Figure 3:**
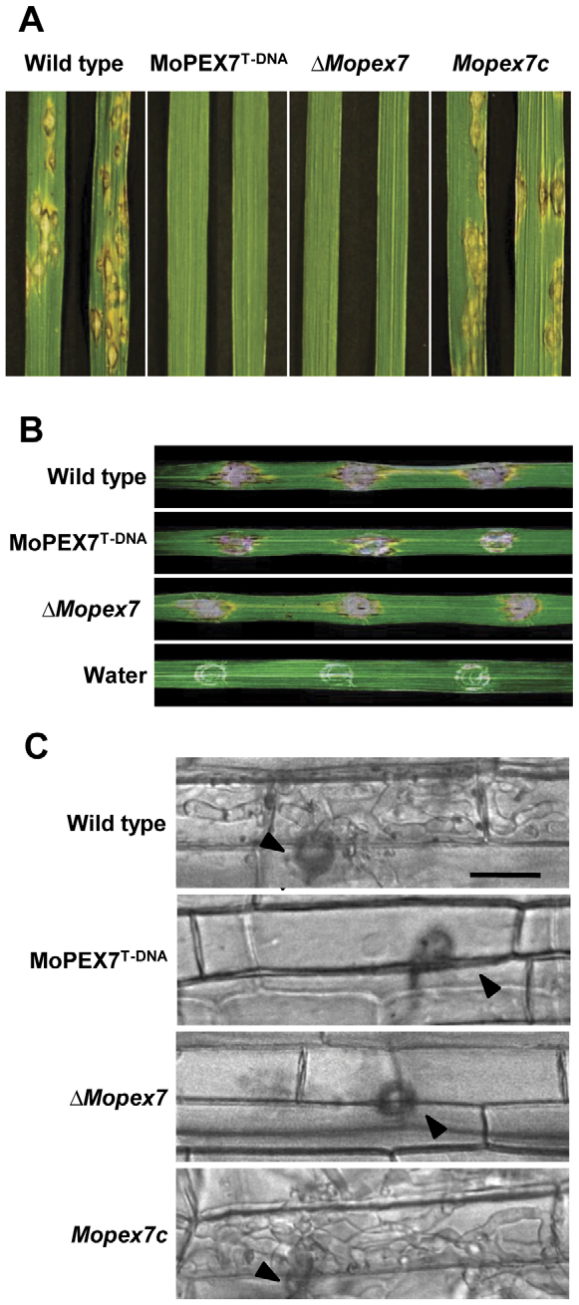
Pathogenicity of *ΔMopex7*. (A) Spray inoculation. Spore concentration was 1×10^5^ conidia/ml. (B) Infiltration inoculation. Conidial suspension was 5×10^4^ conidia/ml. (C) Rice sheath infection 48 h after inoculation. Arrows indicate appressoria. Bar = 20 µm.

**Figure 4:**
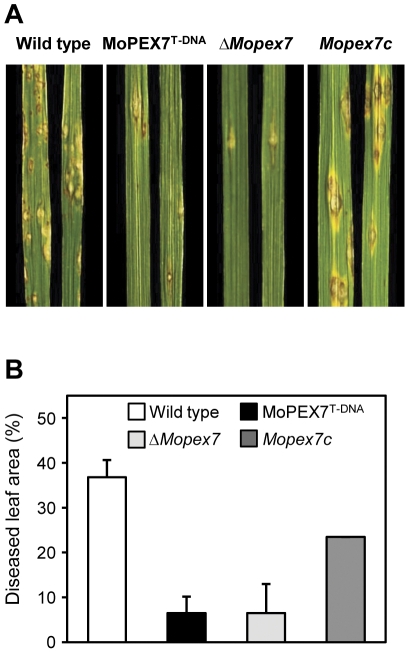
Partial restoration of pathogenicity through addition of glucose. (A) Spray inoculation with 2.5% glucose. Conidial suspension was 1×10^5^ conidia/ml. (B) Quantitative assay of virulence based on diseased leaf area (%).

To investigate the role of MoPEX7 in fungal pathogenicity, conidial suspensions were spray-inoculated onto rice plants of a susceptible cultivar. Our pathogenicity assay showed that, like MoPEX7^T-DNA^, *ΔMopex7* was not able to cause disease on host plants (Figure 3A). The introduction of *MoPEX7* into the deletion mutant restored pathogenicity of the mutant to the wild type level, indicating that *MoPEX7* is indispensable for fungal pathogenicity. Next, we examined *in planta* growth of the mutant by injecting conidial suspension into wound sites, allowing conidia to directly encounter plant tissues without appressorium-mediated penetration. Wound inoculation of the mutant caused as large lesions as in the wild type (Figure 3B). On the rice sheath, the wild type penetrated into plant cells and developed invasive hyphae by 48 h, whereas *ΔMopex7* did not have invasive hyphae (Figure 3C). Notably, additional glucose treatment prior to spray inoculation partially complemented the pathogenicity defect of *ΔMopex7* (Figure 4A). In quantitative analysis of the effect of glucose, disease severity of *ΔMopex7* rose to 5% from 0% (Figure 4B). We further examined appressorial penetration on onion epidermal cells by addition of 1 mM scytalone, an intermediate of melanin or 2.5% glucose (Figure S3). Appressorial penetration defect of *ΔMopex7* was not restored by addition of scytalone, but glucose partially restored penetration defect. It seems that external glucose can be a substitute end product for the PEX7 import system. These results indicate that *ΔMopex7* has a defect in penetration into plant surfaces, but retains the ability to grow inside plant cells.

### MoPEX7 is involved in translocation of lipid droplets and their degradation

**Figure 5:**
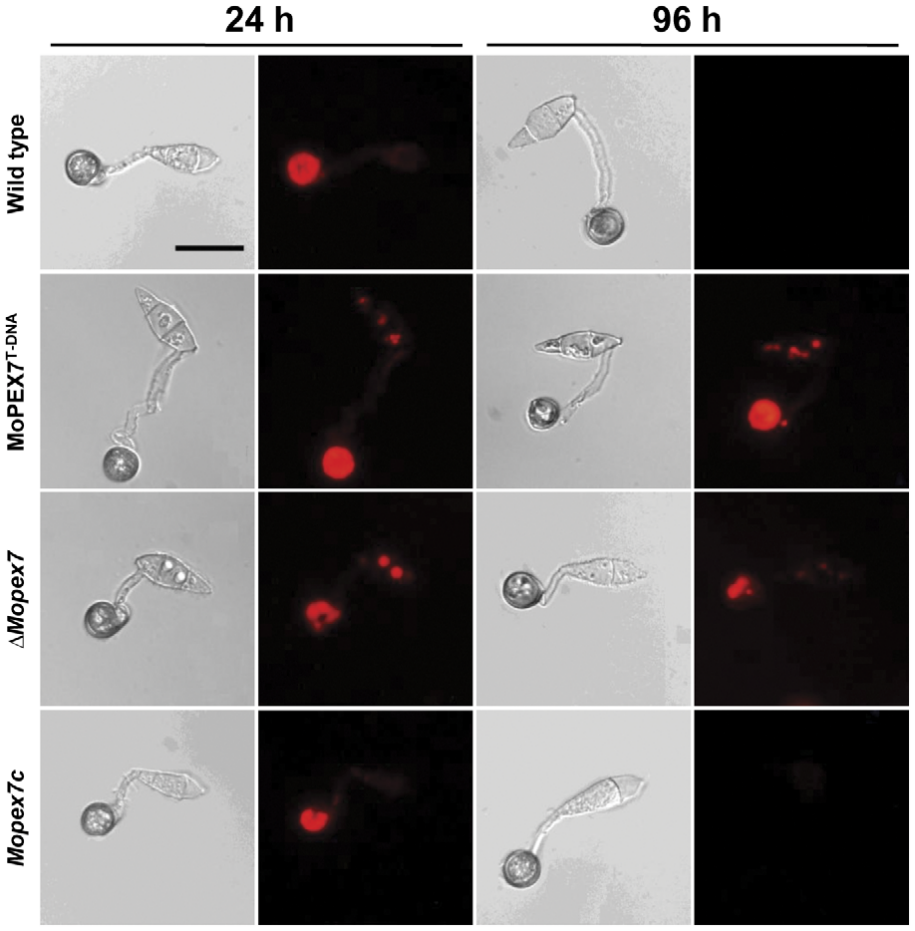
Appressorium morphology and localization of lipid droplets. Cellular localization of lipid droplets was observed after staining with Nile red. Bar = 20 µm.

Lipid translocation and degradation during appressorium development is essential for pathogenicity as a critical part of the turgor generation process [28]. To determine whether MoPEX7 plays a role in lipid mobilization, appressoria of the wild type and mutant were stained with Nile red during appressorium maturation. In the wild type, lipid droplets were completely translocated from conidia to appressoria at 24 h and had degraded at 48 h (Figure 5). In contrast to the wild type, translocation of lipid droplets from conidia to appressoria was delayed and vacuoles in conidia were often observed during appressorium maturation in *ΔMopex7*. Moreover, appressorial lipid droplets of *ΔMopex7* were not degraded even after 96 h. We therefore concluded that MoPEX7 is involved in lipid droplet translocation and degradation.

### MoPEX7 is required for appressorial cell wall porosity

**Figure 6:**
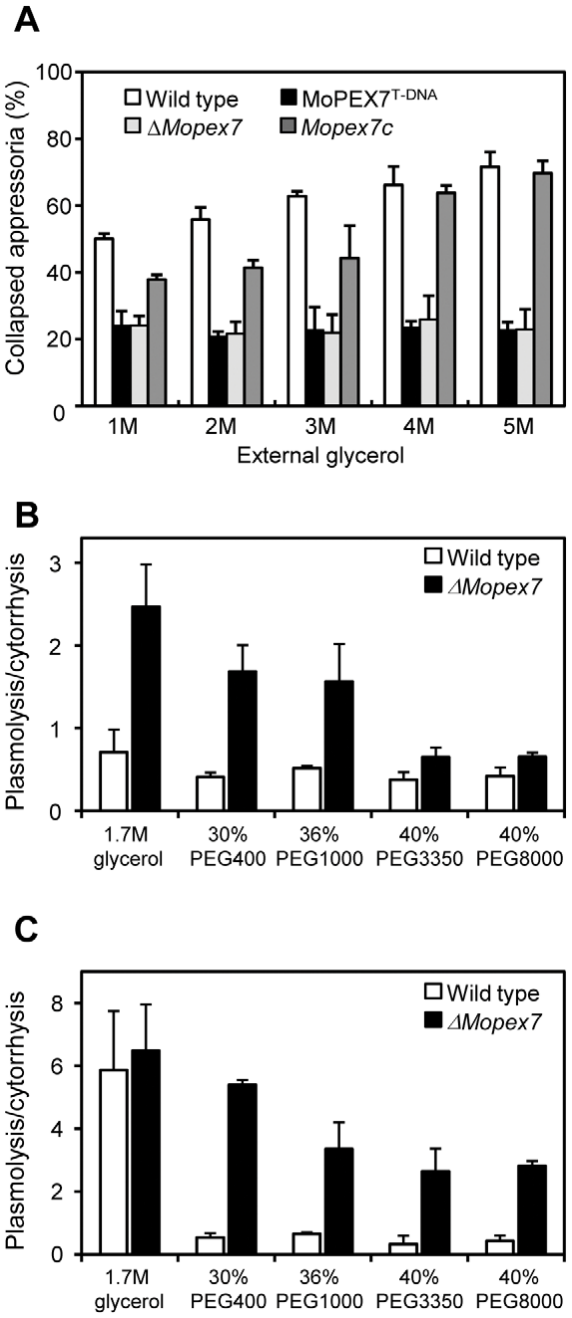
Measurement of turgor pressure and appressorial porosity. (A) Cytorrhysis assay using glycerol at 48 h. (B) Cytorrhysis assay with various osmotic solutions. (C) Cytorrhysis assay with various osmotic solutions after treatment of 4 µg/ml tricyclazole. External osmotic solution was given to appressoria after 48 h. Concentrations were adjusted to 4 MPa osmotic pressure; 1.7 M glycerol, 30% PEG400, 36% PEG1000, 40% PEG3350 and 40% PEG8000 were used as external osmotic solution. Appressorial cytorrhysis and plasmolysis were counted under a microscope. The ratio of plasmolysis to cytorrhysis was the average of three replications.

Lipid degradation during appressorium maturation is connected to turgor generation. Turgor pressure is generated through accumulation of glycerol in the appressorium, and the cellular source of glycerol is glycogen and lipid droplets [19]. Since *ΔMopex7* showed a delay in lipid degradation, we performed a cytorrhysis assay to determine whether *ΔMopex7* is able to generate turgor pressure. Conidia were allowed to form appressoria on plastic coverslips and treated with external glycerol solutions of varying concentration (from 1 M to 5 M). As the concentration of glycerol applied increased, the number of collapsed appressoria increased in the wild type. However, in *ΔMopex7*, fewer appressoria collapsed upon treatment with glycerol than in the wild type (Figure 6A). Two possible reasons may explain these results: higher turgor pressure of mutant appressoria or larger cell wall pores in the mutant than in the wild type. Considering that lipid translocation and degradation of *ΔMopex7* is delayed, one can reasonably rule out higher turgor pressure.

To estimate cell wall pore size, a cytorrhysis assay using polyethylene glycols (PEGs) of different average molecular weight was performed 48 h after appressorium induction. To evaluate the effect of the size of PEG molecules, the concentration of external PEGs was adjusted to produce a constant osmotic pressure of 4 MPa [20], [29]. After a 10-min incubation with PEGs of different molecular weight, cytorrhysis and plasmolysis were observed under a microscope, and the ratio of plasmolysis to cytorrhysis was calculated (Figure 6B). In the wild type strain KJ201, cytorrhysis of appressoia was more frequent over all PEG species tested; in contrast, *ΔMopex7* predominantly showed plasmolysis over cytorrhysis when treated with glycerol, PEG400 and PEG1000. When treated with PEG3350 or higher, *ΔMopex7* showed more cytorrhysis than plasmolysis even though the ratio of cytorrhysis to plasmolysis in the wild type was always less than that of *ΔMopex7*. In addition, *ΔMopex7* had 10% fewer normal appressoria than the wild type after external 1.7 M glycerol treatment (data not shown). Hence, the turgor pressure defect in *ΔMopex7* was caused by both inhibited accumulation of intracellular osmolites and increased appressorial cell wall porosity.

To further understand turgor pressure defect, we also examined appressorial melanization using a melanin biosynthesis inhibitor, tricyclazole, in wild type and *ΔMopex7*. When 1 mM tricyclazole was added in germinating conidia, appressoria of wild type were well melanized but those of *ΔMopex7* were extremely less melanized (Figure S4). Furthermore, we performed cytorrhysis/plasmolysis assays with glycerol or various molecular size of PEGs in the presence of tricyclazole (Figure 6C). When glycerol was treated, appressoria of wild type and *ΔMopex7* showed plasmolysis rather than cytorrhysis. However, when treated with PEGs, wild type and Mopex7 exhibited predominantly cytorrhysis and plasmolysis, respectively.

To assess cell wall integrity, protoplast release was monitored every 30 min after treatment with a cell wall degrading enzyme. No significant difference in protoplast release was observed between the wild type and *ΔMopex7* (Figure S5A). Growth on cell wall perturbing agents like Calcofluor white (CFW) or Congo red (CR) was also not significantly different (*P*<0.05) between the wild type and *ΔMopex7* (Figure S5B). These data suggest that the size of pores in the *ΔMopex7* appressorial cell wall may be larger than in the wild type.

### MoPEX7 is required for utilization of short-chain fatty acids

**Figure 7:**
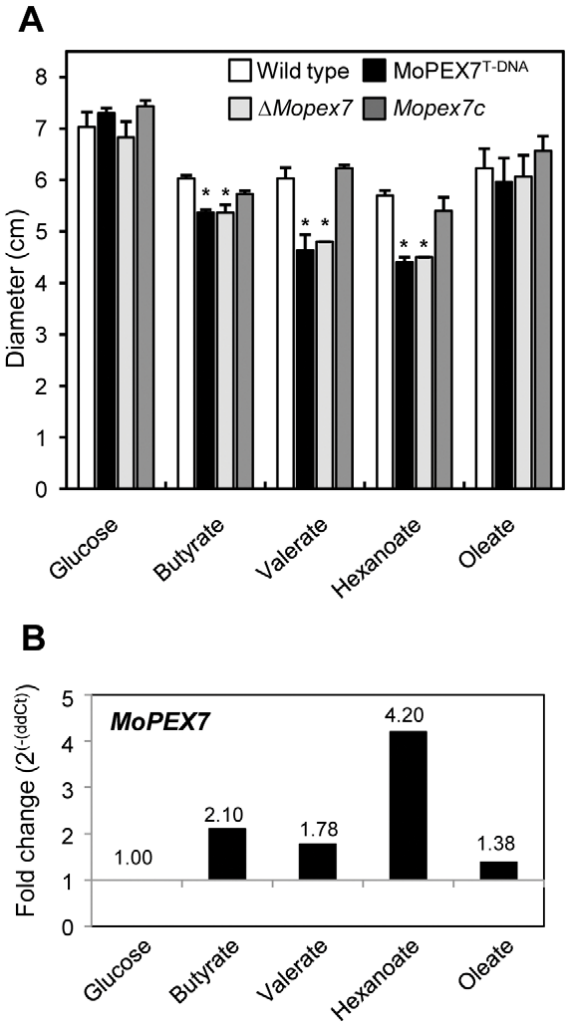
Utilization of fatty acids. (A) Growth on fatty acid media at 12 days post inoculation. Various carbon sources were used as sole carbon source in minimal media: 1% glucose, 2.5 mM oleate, 6 mM butyrate, 5 mM valerate and 4 mM hexanoate. (B) Expression profiling of *MoPEX7* on fatty acid media.

In *S. cerevisiae*, PEX7 is required for fatty acid metabolism, such as that of oleic and lauric acids. In some plant pathogenic fungi, mutants showing a defect in peroxisomal function lose the ability to utilize fatty acids [15], [16], [30]. In *M. oryzae*, MgPEX6 is required for metabolism of fatty acids such as oleic acid and triolein [16], [22]. To assess the contribution of MoPEX7 to fatty acid utilization, *ΔMopex7* was grown on minimal media supplemented with various fatty acids as sole carbon source (Figure 7A). On media containing long-chain fatty acids like oleic acid, no significant difference in *ΔMopex7* growth occurred compared to the wild type. However, growth of *ΔMopex7* significantly decreased on short-chain fatty acid media (*P*<0.05). *ΔMopex7* showed an 11% growth reduction on butyrate (C4), 23% reduction on valerate (C5) and 21% reduction on hexanoate (C6) compared to the wild type. We performed growth tests on other carbon sources (i.e. acetate, Tween20 and olive oil), but *ΔMopex7* growth did not differ from that of the wild type (data not shown). In expression profiling on fatty acid media, *MoPEX7* was more highly expressed on butyrate and hexanoate than on glucose and oleate (Figure 7B). These data suggest that MoPEX7 is involved in utilization of short-chain fatty acids such as butyrate, valerate and hexanoate.

## Discussion

Previous studies on peroxins in phytopathogenic fungi focused on fully abolishing peroxisomal matrix import, so detailed peroxisome-related infection mechanisms remain unclear. In this study, we characterized *MoPEX7* to understand the role of PEX7 involved in translocation of PTS2 proteins, thereby demonstrated that MoPEX7 is required for short-chain fatty acid metabolism and pathogenesis in *M. oryzae*.

Until now, most studies of fungal peroxisomes focused on peroxisomal biogenesis. Among the 31 peroxins reported, most organisms possess different combinations of peroxins. The distribution of peroxins in *M. oryzae* is similar to that in filamentous fungi such as *A. nidulans* and *N. crassa* rather than to that in yeast species [27]. Throughout these organisms, peroxisomal targeting signal receptors have been conserved. PTS protein prediction from a computational analysis is able to discover novel proteins and to understand peroxisomal functions. However, only 73 proteins have been reported as PTS2 candidates among 1126 peroxisomal proteins from 38 organisms in the database, PeroxisomeDB (www.peroxisomedb.org), which is a collection based on known PTS2 protein homology [31]. In spite of expected false-positives because of unclear information on PTS2 sequences and locations, we identified 65 PTS2 candidate proteins *in silico* from regular expression and other location prediction tools. GO term analysis of PTS2 candidates showed that one-third of them are predicted to be involved in metabolism. We characterized *MoTHL1*, one of the PTS2 candidates, whose mutant showed no different phenotype from those of the wild type except for hexanoate use. Moreover, more than half of these PTS2 proteins in *M. oryzae* have yeast homologues, but only seven yeast homologues were predicted to contain PTS2 in the N-terminal. In the case of *A. nidulans*, ten homologues of *M. oryzae* PTS2 candidates have PTS2 at N-termini. This suggests that the fungal PTS2 import system contains species-specific combinations, despite highly conserved PTS2 receptors. Similarly, ICL1 in *S. cerevisiae* is localized in the cytosol while AcuD, an orthologue of ICL1 in *A. nidulans* containing non-typical PTS2, is transferred into peroxisomes by PexG [8]. We consider that the differences in peroxisomal functions in organisms might be due to the composition of PTS proteins. However, in the case of AcuD, PTS2 sometimes shows atypical sequences, and PTS2 candidates from computational analysis dependent on regular expression localize not to peroxisomes but to the cytosol or mitochondria (data not shown). This suggests that other unknown factors apart from the PTS2 sequence could affect peroxisomal matrix protein import.

Beta-oxidation of fatty acids is the principal pathway for fatty acid metabolism in which the end product, acetyl-CoA, can serve as a building block for functions such as energy metabolism and cellular component synthesis. Yeast species only use peroxisomes for fatty acid metabolism, while some filamentous fungi, including *A. nidulans*, use both peroxisomes and mitochondria, and have developed fatty acid degradation mechanisms depending on the length and branching of fatty acids [9], [32]. Complete inhibition of peroxisomal functions such as *pex6* leads to growth reduction on long-chain fatty acids in *C. neoformans*, no growth on long-chain fatty acids in *M. oryzae* and severe growth reduction on both short-chain fatty acids and long-chain fatty acids in *A. Nidulans* [8], [14]. In *A. nidulans* and *P. anserina*, the PTS1 receptor PEX5 is essential for metabolism of long-chain fatty acids, whereas the PTS2 receptor PEX7 assists in such metabolism. *PexG* in *A. nidulans* seems to have specific functions in short-chain fatty acid metabolism. Similar to *A. nidulans*, *ΔMopex7* showed reduced growth only on short-chain fatty acids. This indicates that fungal peroxins have different roles in fatty acid metabolism according to species and form of the fatty acid.

Most peroxisomal metabolism is carried out by matrix proteins. In the case of Fox3 in *S. cerevisiae*, the *MoTHL1* yeast homologue, the *fox3* mutant has severe growth defect on oleate similar to the *pex7* mutant [33]. *MthA*, the *MoTHL1* homologue in *A. nidulans*, is required only for degradation of even-number, short-chain fatty acids. Similar to *mthA*, *ΔMothl1* showed a growth defect only on hexanoate and normal growth on other fatty acids (data not shown). This suggests that peroxisomal function at specific developmental stages or with specific fatty acids is determined not only by peroxisome biogenesis but also through regulation of matrix protein expression.

### Role of the PEX7-mediated import system during pathogenesis in rice blast disease

From studying *MoPEX7*, we demonstrated that peroxisome dysfunction through failure of the PTS2 import system caused loss of virulence on rice. By blocking targeting of PTS2 proteins, *ΔMopex7* showed no disease symptoms on rice leaves. In connection with pathogenicity factors, *ΔMopex7* showed delayed lipid translocation and degradation during appressorium maturation and defects in turgor generation and in plant penetration. Turgor pressure is the physical force required for direct plant penetration, and is necessary for accumulation of intracellular osmolites such as glycerol and substantial outer membrane structures such as the melanin layer and thick cell wall. Cell wall porosity was reported in previous research showing that maintenance of cell wall integrity and biosynthesis of cell wall components are important for pathogenic development of the fungus [20], [34]. In this study, *ΔMopex7* showed defects in not only accumulation of osmolites but also in appressorial melanization and cell wall porosity. Using cytorrhysis/plasmolysis assays with osmolites of different molecular weights and tricyclazole, *ΔMopex7* was found to have larger appressorial cell wall pores than the wild type. However, mycelial cell wall integrity did not seem to be affected in *ΔMopex7*. No differences were observed in the *ΔMopex7* protoplast release assay and mycelial growth with cell wall perturbing agents such as Congo red or Calcofluor white. This defect in appressorial cell wall porosity does not seem to originate from changes in synthesis of major cell wall components like chitin or glucan. Thus, we conclude that MoPEX7 is required for appressorial cell wall porosity. We suggest that MoPEX7 function might be related to the production of minor cell wall components and/or arrangement of cell wall components required for cell wall pore density during appressorium development. In contrast to *ΔMopex7*, complete inhibition of matrix protein import in peroxisomes leads to several problems in melanin biosynthesis, cell wall biosynthesis and woronin bodies in *Δmgpex6* [16], [22]. Moreover, we supposed that PEX5 can function during pathogenesis based on the phenotype difference between *Δmgpex*6 and *ΔMopex7*. Hence, we conclude that appressorium cell wall porosity is the critical factor for turgor generation and pathogenicity.

In the peroxisomal metabolic pathway, genes for fatty acid metabolism have important roles during pathogenesis in fungi causing plant infections [22], [35]. However, peroxisomal fatty acid metabolism in human pathogens is reported to be less important for virulence [36]. The contribution of fatty acid metabolism to pathogenicity in plant pathogenic fungi seems to be crucial for production of acetyl-CoA; it stimulates rapid lipolysis, provides an energy source and is used in making cell wall components, rather than being used simply in turgor generation for penetration [16], [22], [37]. However, MoPEX7 seems to play an essential role for turgor generation rather than in activities such as fatty acid metabolism. Notably, the peroxisome-related mutants, *Δpth2*, *Δclpex6*, *Δmfp1*, *Δmgpex6* and *ΔMopex7*, show partially restored virulence after inoculation of conidia with added glucose [15], [16], [22], [37]. Hence, we consider that glucose addition could be another intermediate of the glyoxylate cycle for energy metabolism, but is not sufficient for complete restoration of pathogenicity.

In this study, we examined the role of peroxisomal metabolism during pathogenesis. The peroxisomal import system through PEX7 is essential for functional appressoria and pathogenicity, independent of the PTS1 import system, in *M. oryzae*.

## Materials and Methods

### Fungal isolates and culture conditions

*M. oryzae* strain KJ201, obtained from the Centre for Fungal Genetic Resources (CFGR; http://cfgr.snu.ac.kr), was used as the wild-type strain. The ATMT0060C3 mutant containing T-DNA insertions was obtained from an *A. tumefaciens*-mediated transformation (ATMT) mutant library in the laboratory of Y.-H. Lee at Seoul National University, Korea [23]. *M. oryzae* strains used in this study were grown on V8 agar [V8; 8% V8 juice (v/v), 1.5% agar (w/v), adjusted to pH 6.0 using NaOH] or oatmeal agar [OMA; 5% oatmeal (w/v), 2% agar (w/v)] at 25°C in constant light to promote conidial production [38]. The fatty acid media was modified minimal media with fatty acids instead of glucose as previously described [39].

### Nucleic acids manipulation and expression analysis

Fungal genomic DNA was isolated by two different methods, depending on future use of the isolated DNA. For Southern DNA hybridization, genomic DNA was isolated from mycelia according to a standard method [40]. For large-scale PCR-based screening of transformants, genomic DNA was isolated as previously described [41]. Southern DNA hybridization was performed on selected transformants to ensure correct gene replacement events and absence of ectopic integration. Genomic DNAs were digested with *Apa*I and blots were probed with 1-kb 5′-flanking sequences of the MoPEX7 locus (Figure 1C). Southern DNA hybridization was performed as previously described [40]. For quantitative real-time PCR, first-strand cDNA was synthesized using the Superscript First-strand Synthesis System III (Invitrogen Life Technologies, Carlsbad, CA, USA) with oligo dT primers from 5 µg RNA. Total RNA was isolated from fresh mycelia cultured in liquid complete media using the Easy-spinTM RNA Extraction Kit (iNtRON Biotechnology, Seongnam, Korea). Quantitative real-time PCR was performed as previously described [42]. and was run on a 7500 Real-Time PCR System (Applied Biosystems, Foster City, CA, USA) using SYBR GREEN PCR Master Mix (Applied Biosystems, Warrington, UK). Primer pairs used are listed in Table 1.

**Table 1:** List of primers in this study.

Name	sequence (5′- 3′)
1481uF	GATGGCTCGCTCCTTTAGA
1481uR	GCACAGGTACACTTGTTTAGAGATGACCAGCCTTACACGATATC
1481dF	CCTTCAATATCATCTTCTGTCGACAAAAATGAAGGATAGTGTTGC
1481dR	CGGCTTTGAATATTTTGTGAT
Hyg B-f	CGACAGAAGATGATATTGAAGG
Hyg B-r	CTCTAAACAAGTGTACCTGTGC
1481RTF2	TATATGCCCTGGGTCTGAAT
1481RTR2	AAGTCGTTCACGCCTAAATC
10700uF	TGAGGCTTGCTGTGATTAAA
10700uR	GCACAGGTACACTTGTTTAGAGAGCGGTACAACGGACTCTAAC
10700dF	CCTTCAATATCATCTTCTGTCGAGGTTTCCTCAGCTGCTTTAG
10700dR	CAGTGCAGTGAGTGTGATGA
10700orfR2	CGCCCTTGCTCACCATCTGCTCATTAACGAACAACC
10700RF	GAAAATTCCAATTTCAGTTGAAC
10700RR	TTAGATGTGATGGTTGAAAGGG
GFP2F	ATGGTGAGCAAGGGC
GFPR	CCAAGCTTATCATCATGCAACATG
SK2223proF	CGGTACCTATAGGGCGAATT
RFP-SKL2	TTATAACTTGGACTTTCTAGATCCG

### Isolation of MoPEX7^T-DNA^, *ΔMopex7*, *ΔMothl1* and rescued strains

The ATMT0060C3 strain was selected from the ATMT mutant library, based on its defective pathogenicity [23]. To determine the genomic location of a T-DNA insertion in ATMT mutants, thermal asymmetric interlaced PCRs (TAIL-PCRs) and inverse PCRs (IPCRs) were performed as previously described [43], [44]. The arbitrary degenerate (AD) primers, border primers, DNA from the T-DNA lines and IPCR primers are listed in Table 1. The amplified PCR products were sequenced at the National Instrumentation Centre for Environmental Management at Seoul National University, and T-DNA insertion sites in the ATMT0060C3 genome were mapped by performing a BLAST search using rescued flanking sequences as queries against the entire *M. oryzae* genome sequence available at the *M. oryzae* genome database at the Broad Institute (http://www.broadinstitute.org/annotation/genome/magnaporthe_grisea/). The *MoPEX7* gene replacement construct was generated using a double-joint PCR (DJ-PCR) method [45]. A total of 1 kb each of 5′- and 3′-flanking DNAs for the MoPEX7 ORF was amplified from genomic DNA. Primers 1481downF and 1481downR were used for the 5′-flanking DNA, and primers 1481upF and 1481upR were for the 3′-flanking DNA (Table 1). The 1.9-kb hph cassette was PCR-amplified from the plasmid pBCATPH [46] using primers Hyg B-r and Hyg_B-f (Table 1). After fusion of the flanking DNAs and the *hph* cassette in PCR, the final construct was amplified using the nested primers 1481upF and 1484downR and used to transform protoplasts obtained from the wild-type strain. Gene deletion of *MoTHL1* was performed in the same way. Protoplasting and transformation of *M. oryzae* was carried out following a standard polyethylene glycol method as described previously [47]. Complementation strains of *ΔMopex7* were made by co-transformation of the pII99 plasmid and fosmid clone FOSKJ_A_23_K02 spanning the *MoPEX7* locus. The transformants were selected based on their ability to grow in the presence of Geneticin® (800 µg/ml) and purified through three to four subcultures on selective media and through single-spore isolation. PCR and Southern DNA hybridization analysis were conducted to confirm replacement and complementation of the *MoPEX7* gene.

### Cellular Localization of MoTHL1::GFP and RFP-SKL fusion protein

The MoTHL1::GFP fusion vector was generated by double-joint PCR [45]. A 2.7-kb PCR product including 1.4 kb of the 5′-flanking promoter region and the full ORF region of the thiolase gene was amplified with the primer pair 10700uF and 10700orf_R (Table 1) from wild type KJ201 genomic DNA. A total of 1 kb of the eGFP ORF including the terminator region was amplified with primer pair GFP2F and EGFP/R (Table 1) from SK2707 (gift from Seogchan Kang, Pennsylvania State University, University Park, PA, USA). The 2.7-kb PCR product of the thiolase gene including the promoter region and the 1-kb PCR product of the eGFP ORF were fused by double-joint PCR. The RFP-SKL construct was amplified with primer pair AsRedF and RFP-SKL2 from SK2233 (gift from Seogchan Kang, Pennsylvania State University). The MoTHL1::GFP fusion construct and RFP-SKL construct were inserted into the pGEM®-T Easy Vector System (Promega, Madison, WI, USA). The GFP fusion vector and RFP fusion vector were introduced into KJ201 and *ΔMopex7* by co-transformation with pII99 carrying the geneticin resistance gene. Cellular localization of MoTHL1::GFP protein in wild type KJ201 and *ΔMopex7* was observed using an Axioplan Universal microscope (Carl Zeiss Microscope Division, Oberkochen, Germany).

### Computational analysis

Homology searches for DNA and protein sequences were performed using BLAST algorithms available at the National Centre for Biotechnology Information (NCBI), Broad Institute and the European Bioinformatics Institute (EBI). Annotation of peroxins in the *M. oryzae* genome was achieved by searching for known peroxins from previous studies, followed by a further BLAST search of the genome [26], [27]. MEGA4 was used for phylogenetic analysis [48]. For identification of the peroxisomal targeting signal two cargo proteins in *M. oryzae*, regular expression analysis using the nonapeptide pattern (R/K)(L/V/I)X_5_(H/Q)(L/A) was performed for the *M. oryzae* 11,074 annotated gene products (http://cfgp.riceblast.snu.ac.kr) [49]. The PTS2 signal was localized within the 50^th^ amino acid from the N-terminal; at the same start time, it had to be 20% of the full-length amino acid sequence from the N-terminal, and the PTS1 signal was assumed to be the 20^th^ amino acid from the C-terminal [50], [51]. Among these candidates, transmembrane proteins, nuclear proteins and secreted proteins were further excluded based on subcellular localization predicted by the TMHMM 2.0, PredictNLS and SignalP programmes.

### Appressorial assay and turgor measurement

Conidia were harvested from 10-day-old cultures on OMA, and conidial suspensions were prepared at a density of 5×10^4^ conidia/ml using sterilized distilled water. Conidial germination and appressorial formation were examined as described by Lee and Dean [52]. Twenty-one droplets of conidial suspensions (5×10^4^ conidia/ml) were placed on microscopic plastic coverslips (Deckglasser, Mülheim, Germany) and incubated in a humid chamber at 25°C. Conidial germination and appressorium formation were measured 24 h post-incubation. Distribution of lipid bodies in germinating conidia and appressoria was examined at 12 h, 24 h, 48 h and 96 h post-incubation with Nile Red staining and epifluorescence microscopy, as described previously [28]. Tricyclazole sensitivity was assesed by measurement of melanized appressoria and non-melanized appressoria at 24 h after dropping spore suspension added tricyclazole on plastic coverslips. Appressorial turgor pressure was also estimated by performing cytorrhysis in various concentrations of osmotic solutions [18], [53]. Mature appressoria 48 h post-incubation were exposed to glycerol or different molecular weights of PEG solution, and the percentage collapsed appressoria and plasmolyzed appressoria was counted under a light microscope [20].

### Penetration assay and pathogenicity assay

Appressorium penetration and invasive growth was observed using rice sheath as previously described [54], [55], [56]. A conidial suspension of 15 µl was dropped on the rice sheath and incubated in a humid chamber at 25°C. Invasive infection hyphae were observed 48 h later with light microscope. A standard plant infection assay was performed by spraying a conidial suspension (10 ml, 1×10^5^ conidia/ml) onto 2-week-old susceptible rice cv. Nakdong. For the infiltration infection assay, 500 µl of conidial suspension (5×10^4^ conidia/ml) was introduced into three leaves with three penetration points per leaf on 3-week-old rice. Penetration ratio was observed on onion surface at 36 h after dropping conidial suspension (5×10^4^ conidia/ml) with 1% glucose or 1 mM scytalone. The plants or leaves were examined for infection symptoms 7 days post-inoculation. The disease severity was assessed from the percentage diseased leaf area as calculated using the Axiovision image analyzer [57].

### Cell wall integrity measurement

Cell wall integrity was assessed by either examining the sensitivity of fungus to cell wall degrading enzyme and cell wall stress agents, or measuring FT-IR profile. Protoplast production was performed by previously descript [20]. Cell wall integrity was measured using growth on complete media with cell wall perturbing agent Calcofluor white (200 ppm) or Congo red (200 ppm).

## Supporting Information

Figure S1 **Identification of MoPEX7^T-DNA^.** (A) T-DNA insertion information of ATMT0060C3 from TAIL-PCR. (B) Southern DNA hybridization analysis of ATMT0060C3 (MoPEX7^T-DNA^). Genomic DNA was digested with *Kpn*I (1,2), *Apa*I (3,4), *Sca*I(5,6) and *Xho*I (7,8). The *hph* cassette was used as probe. Lanes 1, 3, 5 and 7 are wild-type strain KJ201. Lanes 2, 4, 6 and 8 are MoPEX7^T-DNA^.
(TIF)

Figure S2 **Phylogenetic analysis of PEX7 in fungi.**
(TIF)

Figure S3 **Penetration ratio of** ***ΔMopex7*** **with addition of 2.5% glucose or 1 mM scytalone on onion epidermal surface at 36 h.**
(TIF)

Figure S4 **Tricyclazole sensitivity of** ***ΔMopex7*** **.** Melanized appressoria ratio was observed at 24 h under a light microscope.
(TIF)

Figure S5 **Cell wall integrity of** ***ΔMopex7*** **.** (A) Protoplast production from cell wall degrading enzyme treatment. (B) Growth on complete media with cell wall synthesis inhibitor at 8 days. A total of 200 µg/ml Calcofluor white (CFW) or Congo red (CR) was added to complete media (control).
(TIF)

Table S1 **List of putative peroxins in** ***M. oryzae*** **.**
(XLS)

Table S2 **List of putative PTS2 cargo proteins in** ***M. oryzae*** **.**
(XLS)
