# Supplementary figures and images for: The PEX7-Mediated Peroxisomal Import System Is Required for Fungal Development and Pathogenicity in Magnaporthe oryzae
Source: PLoS One. 2011 Dec 14;6(12):e28220. doi: 10.1371/journal.pone.0028220 (PMC3237427; doi:10.1371/journal.pone.0028220)

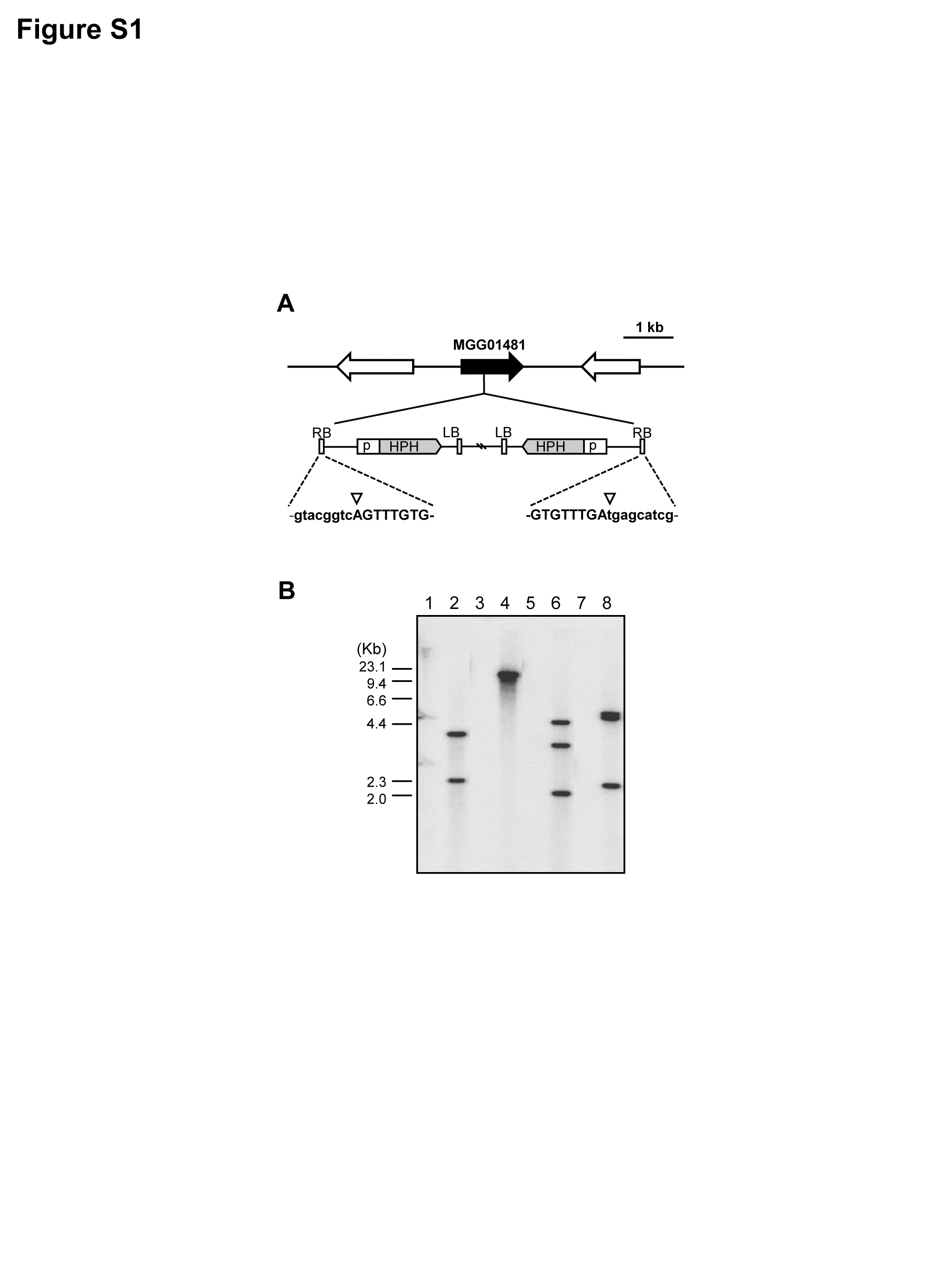

Supplement: Figure S1 — Identification of MoPEX7T-DNA. (A) T-DNA insertion information of ATMT0060C3 from TAIL-PCR. (B) Southern DNA hybridization analysis of ATMT0060C3 (MoPEX7T-DNA). Genomic DNA was digested with KpnI (1,2), ApaI (3,4), ScaI(5,6) and XhoI (7,8). The hph cassette was used as probe. Lanes 1, 3, 5 and 7 are wild-type strain KJ201. Lanes 2, 4, 6 and 8 are MoPEX7T-DNA. (TIF) [file pone.0028220.s001.tif]

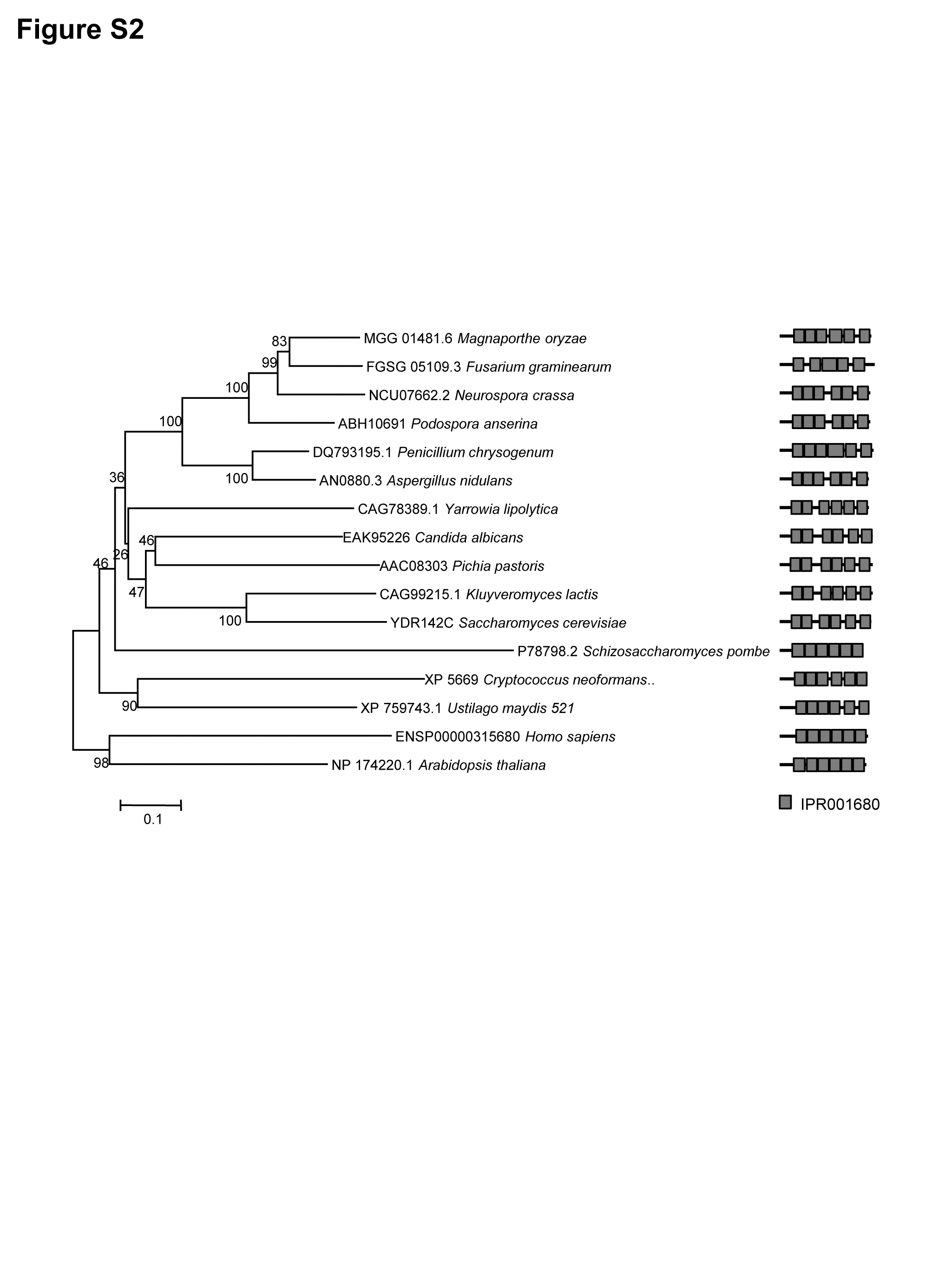

Supplement: Figure S2 — Phylogenetic analysis of PEX7 in fungi. (TIF) [file pone.0028220.s002.tif]

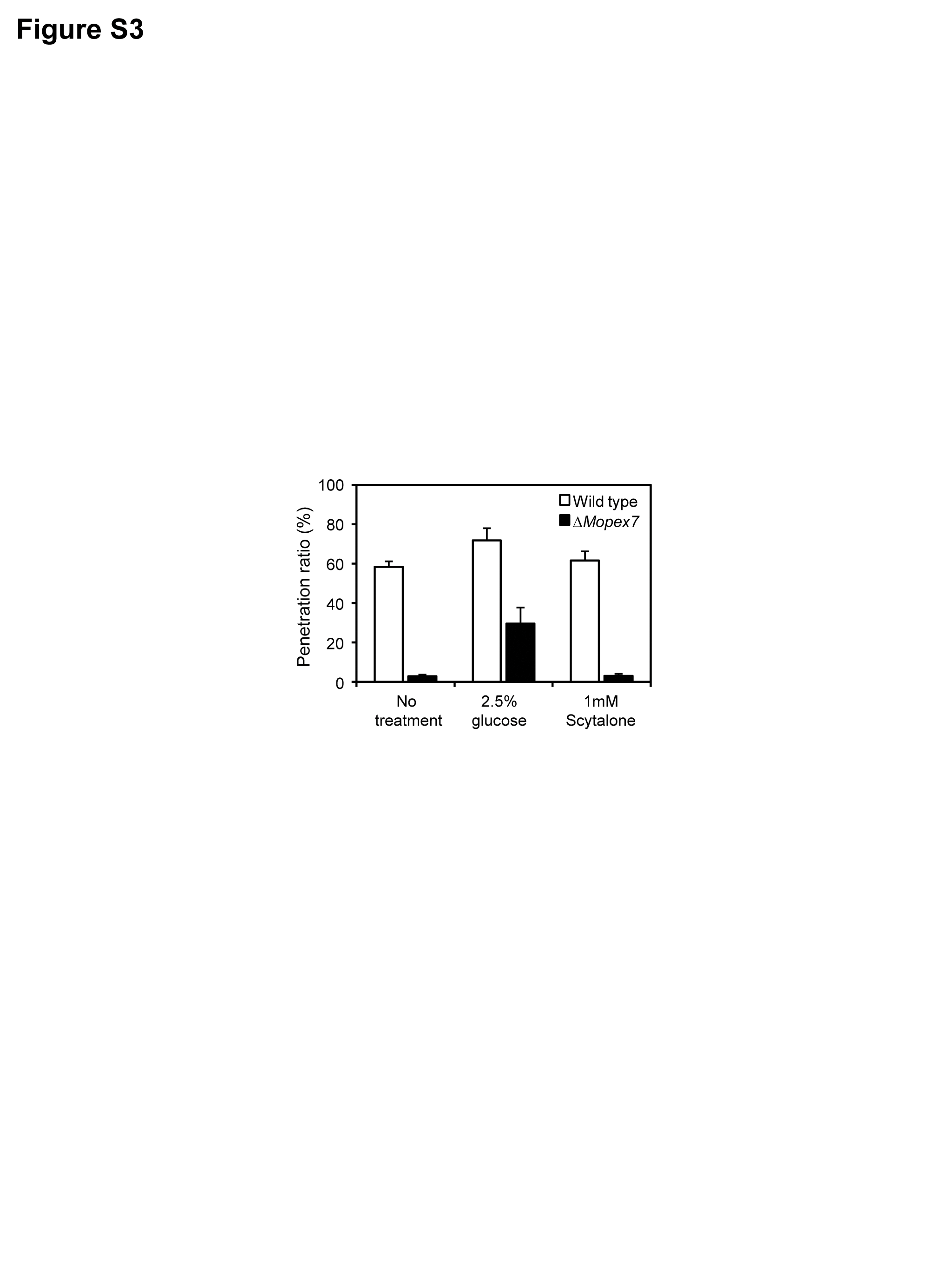

Supplement: Figure S3 — Penetration ratio of ΔMopex7 with addition of 2.5% glucose or 1 mM scytalone on onion epidermal surface at 36 h. (TIF) [file pone.0028220.s003.tif]

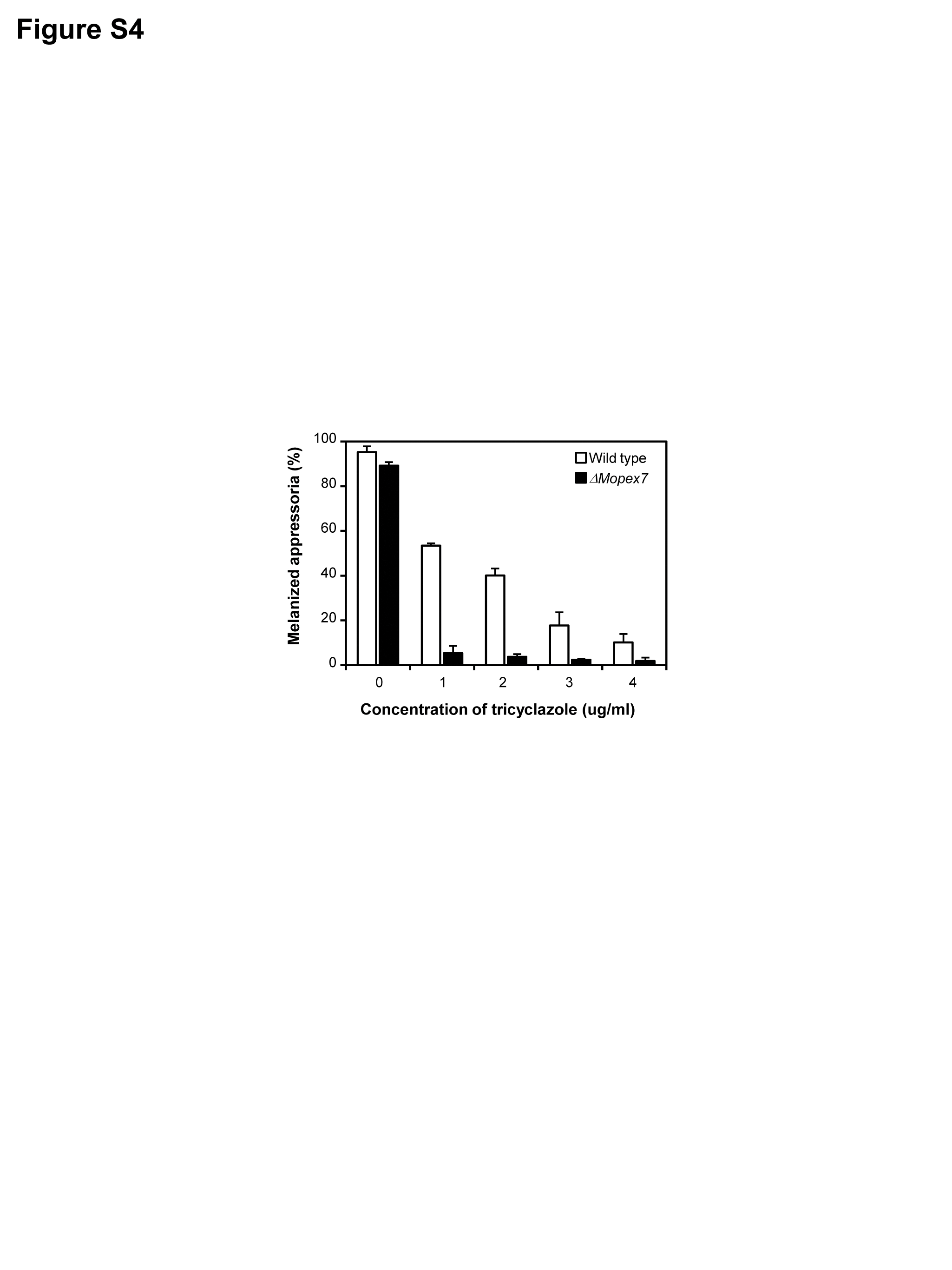

Supplement: Figure S4 — Tricyclazole sensitivity of ΔMopex7 . Melanized appressoria ratio was observed at 24 h under a light microscope. (TIF) [file pone.0028220.s004.tif]

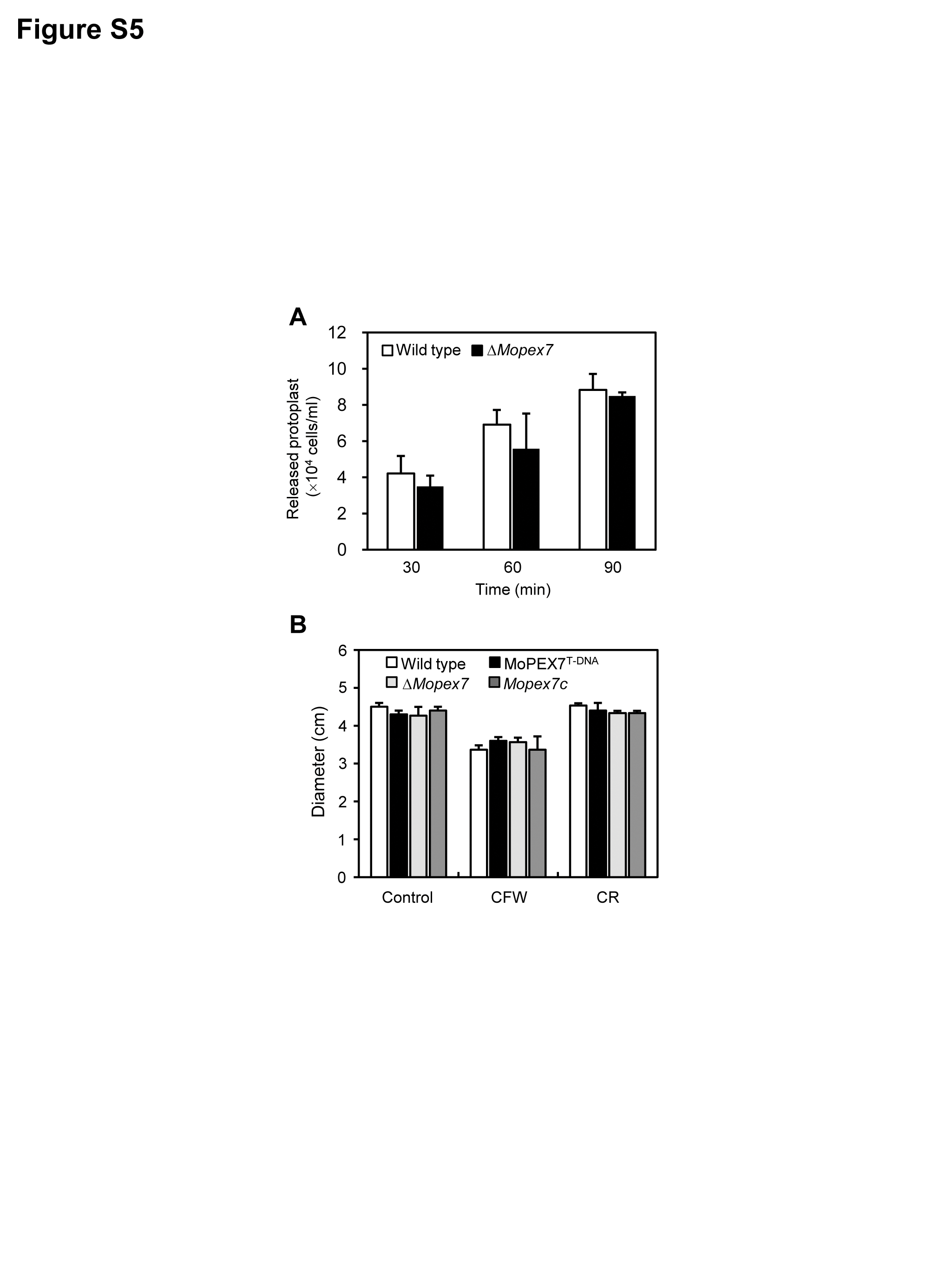

Supplement: Figure S5 — Cell wall integrity of ΔMopex7 . (A) Protoplast production from cell wall degrading enzyme treatment. (B) Growth on complete media with cell wall synthesis inhibitor at 8 days. A total of 200 µg/ml Calcofluor white (CFW) or Congo red (CR) was added to complete media (control). (TIF) [file pone.0028220.s005.tif]
